# Understanding the strengths and limitations of recombinant protein studies

**DOI:** 10.3389/fmolb.2026.1927411

**Published:** 2026-09-29

**Authors:** Daniele Dell’Orco, Karl-Wilhelm Koch

**Affiliations:** 1 Department of Neurosciences, Biomedicine and Movement Sciences, Section of Biological Chemistry, University of Verona, Verona, Italy; 2 Department of Neuroscience, Biochemistry Group, Carl von Ossietzky Universität Oldenburg, Oldenburg, Germany

**Keywords:** protein analytics, protein expression, protein folding, protein interaction studies, three-dimensional structure

## Abstract

Recombinant proteins are the workhorse of modern biochemical research, yet their use *in vitro* is routinely questioned on grounds of physiological relevance. Here we provide a structured guide to the strengths and limitations of recombinant protein studies, framed within the broader contrast between systemic (top-down) and reductionist (bottom-up) approaches to biological questions. We examine the main sources of concern. These are incorrect or incomplete folding, missing or aberrant post-translational modifications, the absence of native cofactors, the consequences of purification, and the lack of cellular crowding. Each of them is an experimentally measurable property rather than an assumption to be conceded. Against these we set the corresponding strengths: molecular-level resolution, well-defined interaction studies, and access to biophysical techniques that require purified material. The guide is organized around three practical tools, designed to be used in sequence: a comparison of expression hosts, a minimum checklist for validating a preparation before quantitative use, and a troubleshooting table for when a check fails. We argue that recombinant proteins are best understood not as a substitute for physiological studies but as a bridge between *in silico*, *in vitro*, and *in vivo* research.

## Introduction

Recombinant DNA technology changed fundamentally the investigation of protein structure and function. It allows proteins to be expressed in large amounts, to combine proteins with so-called fusion domains, to create chimeric proteins or to introduce mutations (Structural Genomics Consortium et al., 2008; Rosano and Ceccarelli, 2014; Schütz et al., 2023; de Marco, 2025). As a more recent achievement, computer-assisted protein design allows creating new protein folds with new properties (Lovelock et al., 2022; Yang et al., 2026). However, using recombinant proteins *in vitro* to address questions of structure-related properties raises concerns about biological or physiological relevance. In our own experience, these concerns are raised repeatedly in discussions with colleagues, in the comments of referees and editors during peer review, and in PhD defenses. Motivated by these discussions, we collect here the arguments for and against the use of recombinant proteins in biological research.

Two essentially opposite approaches can be used for working on a biological question: *systemic* and *pure reductionist* (Figure 1). We will first describe briefly these two approaches before we discuss the different arguments and justifications of using one or the other.

**FIGURE 1:**
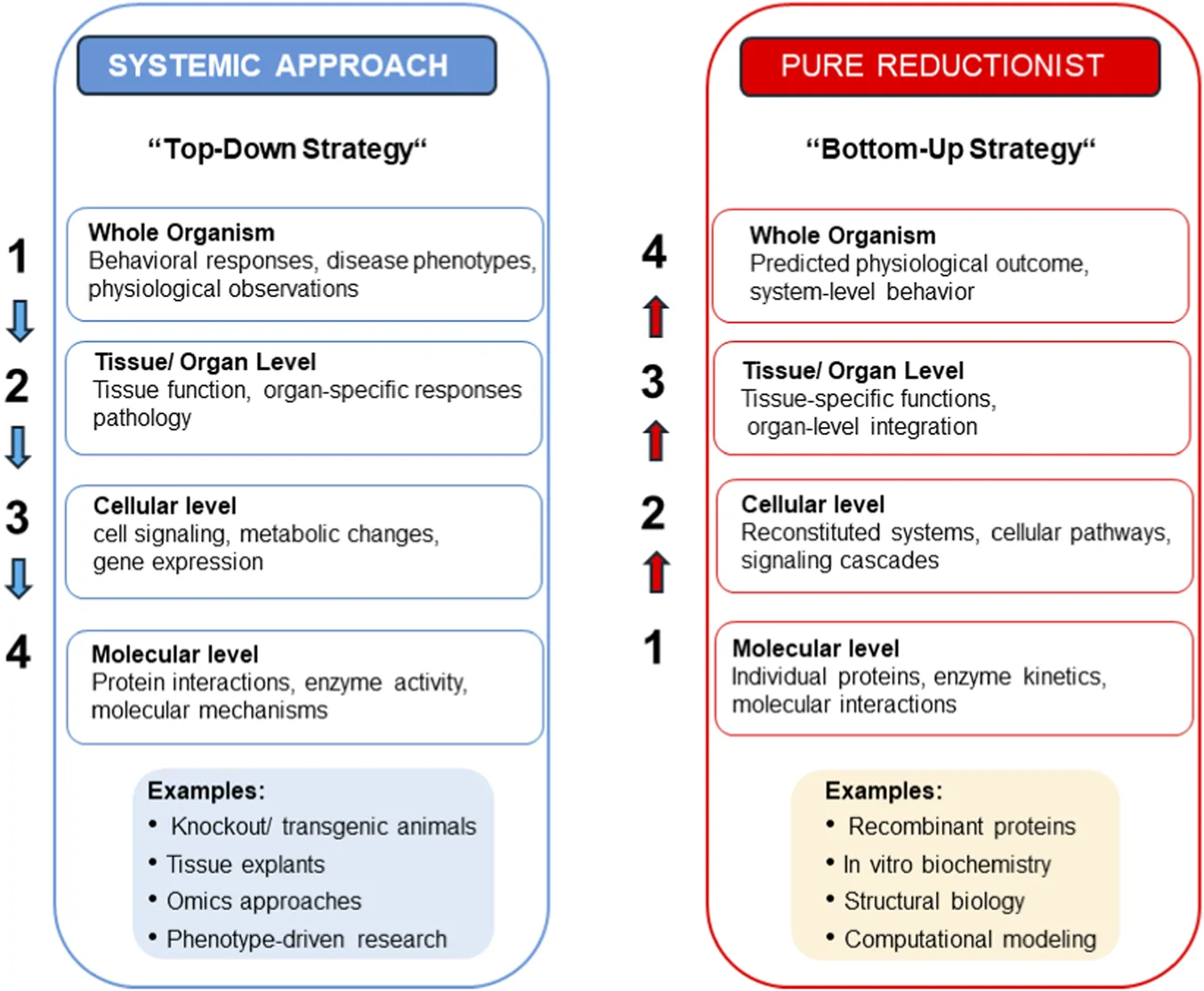
Comparison of systemic versus pure reductionist approaches to investigate biological questions. The two columns represent the same biological problem approached from opposite directions. In the systemic, top-down strategy (blue, left) investigation begins at the level of the whole organism and proceeds downwards through tissue and organ, cellular and molecular levels. In the pure reductionist, bottom-up strategy (red, right) it begins with individual molecules and proceeds upwards towards the predicted behaviour of the system. The levels are numbered 1 to 4 in the order in which each strategy traverses them, and the vertical arrows indicate the direction in which each strategy proceeds; the two directions are complementary rather than mutually exclusive. The boxes at the bottom of each column list representative experimental approaches associated with each strategy.

### Systemic approaches

The genomes of an increasing number of species are now available, both of classic model organisms such as *Drosophila* and mice and of species that have not so far served as biological models. This availability brings into focus the knowledge gap between genetic information and protein function. A direct way to explore a possible function is to create a genetically modified organism (Evans, 2001; Capecchi, 2001; Palmiter and Brinster, 1985; Maeda and Smithies, 1986). In such an organism the gene of interest is no longer expressed (knock out), or is expressed in a modified form after the introduction of a mutation (knock-in). Alternatively, a gene from a foreign organism is deliberately inserted into the host genome (transgenic animal). In some cases the general function of a protein is known, but the impact of a modification such as a disease-causing mutation is not. A genetically modified animal, together with studies of its behaviour and physiology, can then give important insights into genetically caused dysfunction.

A biological process studied directly in the context of a whole organism or a tissue directly extracted from the animal underlies the spreading of “top-down” approaches. In these, research begins with the whole organism, tissue or biological system and progressively narrows down to individual components, such as specific proteins. Research steps typically start by observing a physiological process, phenotype, or disease state at the macroscopic or tissue level. From there, experimental techniques (e.g., gene knockout, pharmacological inhibition, *omics* analyses) usually allow the identification of the molecular players involved in the process. The systemic approach in principle allows an investigation of how a specific protein contributes to the overall biological outcome and to the observed phenotypes, including behavioural responses. Isolating the specific contribution of a single protein or pathway can nevertheless be difficult. Many interacting variables—such as compensatory mechanisms, environmental factors, or cell-to-cell variability—can mask or confound the function of the target protein.

### Pure reductionist approaches

The advent of recombinant DNA technology has greatly accelerated the rise of reductionist biological science, enabling the detailed study of individual molecules involved in specific biological pathways. Using the techniques outlined below, this approach allows researchers to break down biological complexity into fundamental molecular components. A deep understanding of these building blocks can, in principle, provide insights that are broadly applicable to many other, seemingly unrelated biological processes. This has led to the so called “bottom-up” approach, which begins at the molecular level, typically by studying individual proteins or isolated molecular components in controlled environments (*in vitro*). Researchers analyze a protein’s structure, interactions, and biochemical properties to understand how it functions and contributes to broader biological processes. On the other hand, the reductionist approach can oversimplify complex biological systems by isolating components from their context, limiting our understanding of emergent behaviours and system-level regulation.

Throughout this article we take the view that systemic and reductionist strategies are not competing alternatives. They are complementary descriptions of the same multifactorial problem, each valid within the boundaries of the system in which the data are generated. *In vitro* measurements describe the behaviour of a defined molecular system under controlled conditions. Relating them to the behaviour of a cell or an organism requires an extrapolation whose magnitude is proportional to the simplification introduced. Conversely, observations made at the level of the organism integrate a large number of variables and rarely resolve the contribution of a single molecular event. The two levels are therefore best regarded as distinct but semi-dependent states of the same biological problem. The decision on which approach to adopt, or more often on how to combine them, should be made case by case, on the basis of the question being asked.

In this tutorial-intended review article, we focus on one fundamental component of biological processes, namely proteins. We discuss the advantages and disadvantages of using recombinant proteins as a tool to bridge the seemingly separate worlds of systemic and reductionist approaches. The box in Figure 2 summarizes the main steps necessary to produce recombinant proteins. Methodological details are out of the scope of this article, and the reader can refer to specific literature (Structural Genomics Consortium et al., 2008; Rosano and Ceccarelli, 2014; Schütz et al., 2023; de Marco, 2025; Dash et al., 2023; Subedi et al., 2015; Oliveira and Domingues, 2018; Errey and Fiez-Vandal, 2020). Parallel *in vitro* studies using recombinant proteins sometimes end up in a mismatch of results. This gives rise to a list of arguments against the use of recombinant proteins, or to questions about the validity of the conclusions drawn in such studies. Some of the main arguments are discussed below and summarized in Table 1. Beyond this discussion, the guide is organized around three practical tools that the reader can consult directly. Table 2 compares the available expression hosts and the modifications each of them can deliver. Table 3 is a minimum checklist of the properties that a recombinant preparation should be shown to possess before it is used quantitatively. Table 4 collects common failure modes with their probable causes and the measurements that discriminate among them.

**FIGURE 2:**
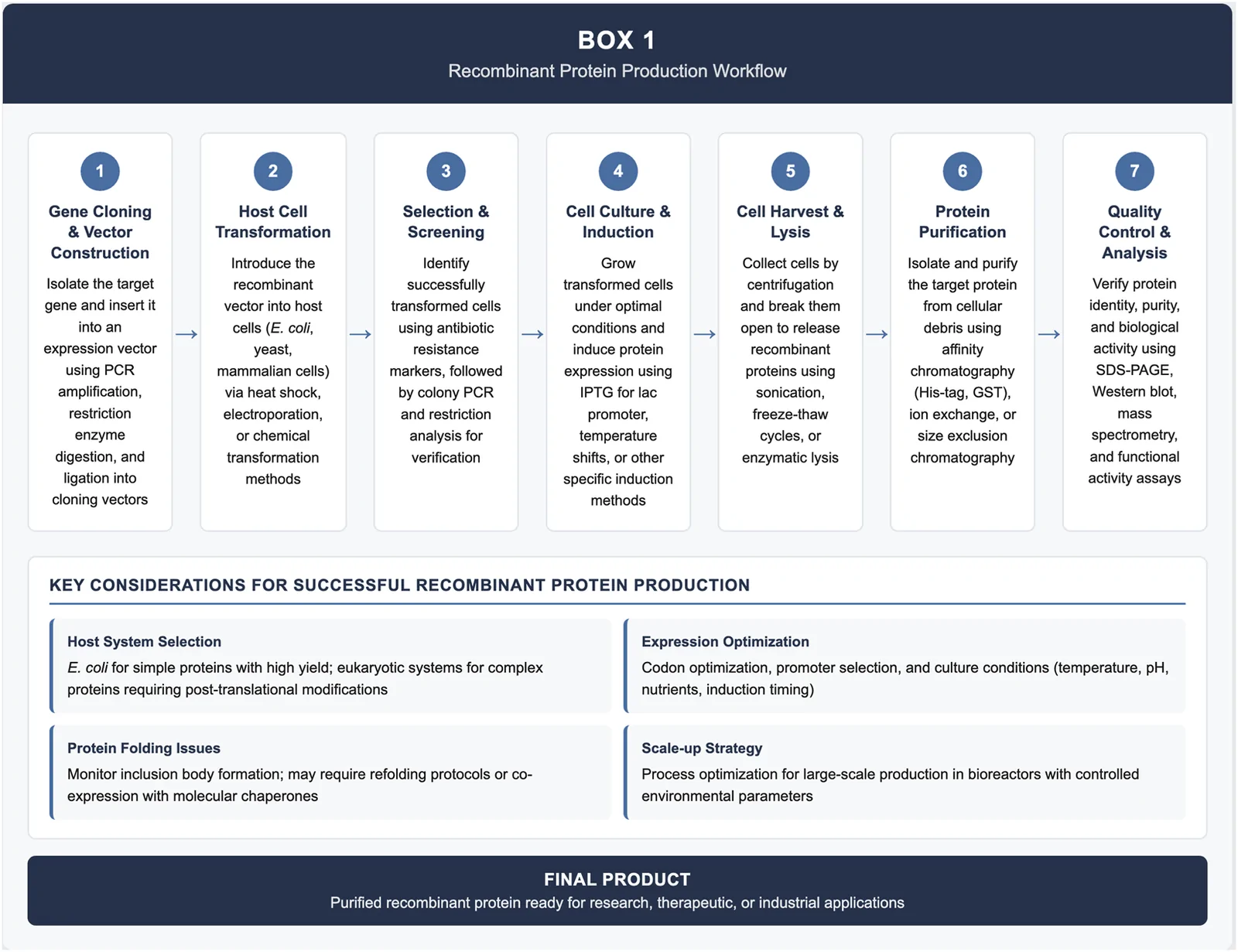
Workflow for the production of recombinant proteins. The seven numbered steps summarize the route from the target gene to the purified product. The first steps are cloning of the gene into an expression vector, transformation of the host cell, and selection and screening of transformants. The remaining steps are culture and induction of expression, harvest and lysis of the cells, chromatographic purification, and quality control of the final material. The lower panel collects the four decisions that most often determine whether the workflow succeeds. These are the choice of host system, the optimization of expression conditions, the management of folding problems, and the strategy for scale-up. Methodological details are beyond the scope of this article and are discussed in the references given in the text; the choice of host is developed in Table 2, and the quality-control step in Table 3.

**TABLE 1:** Strengths and limitations of working with recombinant proteins. Each entry gives a short statement followed by a brief explanation. The topics are developed in the corresponding sections of the text, while the practical consequences for the choice of expression host, for validation, and for troubleshooting are collected in Tables 2–4.

*Strengths* of working with recombinant proteins	*Limitations* of working with recombinant proteins
High availability and scalability. Can be produced in relatively large quantities once an expression system is established	Non-native expression environment. The protein may not experience the same cellular environment, cofactors, or quality-control mechanisms as in its native context
Experimental control. Expression conditions, protein sequence, concentration, and purification can be controlled	Folding and stability issues. Recombinant proteins may misfold, aggregate, or have altered stability compared with the native protein
Protein engineering. Tags, mutations, truncations, or specific modifications can be introduced to facilitate purification and detection, or to test structure-function hypotheses	Missing or aberrant post-translational modifications. Prokaryotic hosts cannot glycosylate, and other hosts install glycan structures that differ from the human ones; function may depend on them
Access to non-native chemistries. Unnatural amino acids, site-specific labels and defined chemical modifications can be installed that no native source provides	Effects of affinity tags. Tags such as His6, GST, FLAG or Fc can influence protein structure, stability, oligomerization, or interactions with binding partners
Defined composition. The purified preparation can be characterized and used under controlled conditions, allowing detailed biophysical and structural studies	Heterogeneity. Different conformational states, oligomers, glycoforms, or processing states may coexist in the purified sample
Reproducibility. A defined recombinant construct can be produced repeatedly under standardized conditions	Loss of native interactions. Purification removes the protein from its native cellular environment and can disrupt complexes or transient interactions
Compatibility with many analytical techniques. Suitable for structural, biophysical, enzymatic, and binding assays	Expression-system-specific artifacts. Bacterial, yeast, insect, or mammalian expression systems can each introduce distinct biochemical properties
Facilitates structure-function studies. Mutants and engineered variants can be compared systematically, giving insight into function at the molecular level	Concentration artifacts. Protein concentrations used *in vitro* may be substantially higher than physiological concentrations, potentially promoting non-native interactions or oligomerization
Well suited for screening. Purified proteins can be used in high-throughput biochemical or biophysical assays	May not fully represent physiological behaviour. Results obtained with isolated recombinant protein may not directly reflect the native cellular context

**TABLE 2:** Comparison of expression systems for recombinant protein production. Yields are indicative orders of magnitude for research-scale work and vary widely with the target protein and the degree of optimization. Times refer to the interval between an available expression construct and the first purified material. The table is intended as a guide to the choice of host rather than as a specification of any individual process.

Host	Typical yield and time to protein	Disulfide bonds	N-glycosylation	Other PTMs	Relative cost	Typical use	Main limitation
*E. coli*, standard (BL21(DE3) and derivatives)	10–100 mg/L, up to 100–800 mg/L optimized, g/L in bioreactors; days	Not in the cytoplasm; possible via periplasmic targeting	None	None natively; some installable by co-expression of the modifying enzyme	Lowest	Soluble cytosolic proteins, mutant panels, isotope labelling	No eukaryotic modification machinery; inclusion bodies; endotoxin
*E. coli*, oxidizing cytoplasm (Origami, SHuffle)	mg/L to tens of mg/L; days	Yes, in the cytoplasm	None	As above	Low	Disulfide-containing proteins without refolding	Reduced growth rate and yield
*E. coli*, glycoengineered	Low and strongly construct-dependent; weeks	As parent strain	Bacterial-type N-glycans	As above	Moderate	Proof-of-concept glycoprotein work	Glycan structures differ from eukaryotic ones; specialized
Yeast (*P. pastoris*, *S. cerevisiae*)	Tens to hundreds of mg/L, often secreted; 2–4 weeks	Yes	Yes, but high-mannose and prone to hypermannosylation	O-mannosylation; most others	Low to moderate	Secreted proteins where glycan identity is not critical	Non-human glycan structures unless glycoengineered
Insect cells/baculovirus	1–50 mg/L; 4–8 weeks including virus generation	Yes	Yes, typically paucimannosidic	Most, including phosphorylation and lipidation	Moderate	Multiprotein complexes, kinases, membrane proteins	Long lead time; glycans truncated relative to mammalian
HEK293, transient transfection	1–50 mg/L, up to about 100 mg/L in optimized suspension culture; 1–2 weeks	Yes	Yes, human-type	Full mammalian repertoire	High	Human secreted and membrane proteins; rapid supply of correctly modified material	Cost; scale limited relative to stable lines
CHO, stable cell line	g/L in fed-batch; months to establish the line	Yes	Yes, human-like	Full mammalian repertoire	Highest	Therapeutic glycoproteins; consistent supply across lots	Development time incompatible with exploratory work
Cell-free, prokaryotic lysate	Up to g/L in optimized systems; hours to days	Yes, with adjusted redox conditions	None	Limited; unnatural amino acids readily incorporated	High per mg	Toxic proteins, membrane proteins in nanodiscs, parallel screening of variants	Cost per milligram; modification capacity limited to the lysate
Cell-free, eukaryotic lysate	Lower than prokaryotic systems; hours to days	Yes	Possible when microsomes are supplied	Broader than prokaryotic lysates	Highest per mg	Targets requiring eukaryotic processing at small scale	Yield and cost restrict it to analytical amounts

**TABLE 3:** Minimum characterization of a recombinant protein preparation before quantitative use. No single entry is sufficient on its own, and none replaces a functional assay. The order given is the order in which the checks are best applied; failure at any point should send the preparation back to the expression or purification stage (Table 4) rather than into the measurement.

Property	Question	Method	Readout	Interpretation
Identity	Is this the intended protein?	Intact-mass MS; peptide mapping by LC-MS/MS	Measured versus calculated mass; sequence coverage	Mass deviation indicates truncation, incomplete tag cleavage or an unexpected modification
Purity	What else is in the sample?	SDS-PAGE; reverse-phase HPLC	Band pattern; peak area	A single band on 1D SDS-PAGE is necessary but not sufficient
Homogeneity and oligomeric state	Is it one species, and which?	SEC-MALS; analytical ultracentrifugation; DLS	Absolute mass in solution; sedimentation coefficient distribution	Soluble oligomers and aggregates invisible on a gel are resolved here
Active concentration	How much of it works?	Functional assay against a standard; active-site titration	Specific activity	The denominator of every kinetic parameter; an error here propagates proportionally
Secondary structure	Is it folded?	Far-UV circular dichroism	Spectral shape; helix and sheet content	Loss of the characteristic minima indicates unfolded or aggregated material
Tertiary fold	Is it well packed?	Near-UV circular dichroism; intrinsic tryptophan fluorescence	Aromatic fine structure; emission maximum	A red-shifted emission maximum indicates solvent-exposed tryptophans
Thermal stability	How robust is it, and does the ligand stabilize it?	DSC; nanoDSF; thermal shift assay; thermal denaturation by CD	Melting temperature; transition enthalpy and cooperativity	A broad or multiphasic transition suggests a heterogeneous or partly unfolded population
Posttranslational modifications	Present, where, and on what fraction?	LC-MS/MS peptide mapping; released-glycan analysis	Site localization; site occupancy	Partial occupancy means the sample is a mixture, with consequences for stoichiometry
Cofactors and metals	Is the cofactor there?	ICP-MS; UV-visible or EPR spectroscopy; reconstitution assay	Stoichiometry per monomer	Sub-stoichiometric loading mimics reduced activity
Batch consistency	Is this batch the same as the last?	Repetition of the above on each preparation	Comparison against a reference batch	Drift between batches is a frequent and under-reported source of irreproducibility

**TABLE 4:** Common failure modes in the production and purification of recombinant proteins, with probable causes, interventions and the measurement that discriminates among them. Entries are ordered approximately along the workflow of Figure 2, from expression through purification to the biophysical measurement.

Observation	Probable cause	Intervention	Diagnostic check
No detectable expression	Rare codons; mRNA instability; product toxicity; promoter leakage	Codon-optimized construct or a tRNA-supplementing strain; tighter promoter control; cell-free expression	Compare induced and uninduced whole-cell lysates by SDS-PAGE and Western blot
Product entirely in inclusion bodies	Folding rate exceeded by synthesis rate; missing disulfides; exposed hydrophobic surfaces	Lower induction temperature and inducer concentration; co-express chaperones; periplasmic targeting; solubility tag; refolding from denaturant as a last resort	Fractionate soluble and insoluble material in parallel at each condition
Soluble but inactive	Kinetically trapped conformer; missing cofactor or PTM; incorrect disulfide pairing	Change host; supply the cofactor during or after expression; oxidizing-cytoplasm strain	Far-UV circular dichroism and thermal denaturation compared with a reference preparation
Aggregation during or after purification	Concentration above the solubility limit; buffer far from optimum; freeze-thaw damage	Buffer and additive screening by nanoDSF; concentrate later and less; avoid repeated freezing	SEC-MALS or DLS before and after each step
Progressive degradation	Endogenous proteases; exposed flexible loops	Protease inhibitors; lower temperature; shorten the protocol; redesign construct boundaries	Intact-mass MS at successive time points
Heterogeneous mass by MS	Incomplete tag cleavage; partial PTM occupancy; N-terminal methionine processing; oxidation	Optimize cleavage conditions; change host to one delivering the required modification	Peptide mapping to localize the source of heterogeneity
Loss of cofactor or metal	Chelators in the buffer; cofactor incorporated only co-translationally	Remove chelators; supplement during expression; change host	ICP-MS or a spectroscopic signature specific to the holo-form
Tag interferes with the assay	Occlusion of the interface; altered oligomeric state; intrinsic signal of the tag	Cleave the tag; move it to the other terminus; lengthen the linker	Compare tagged and untagged preparations in the same assay
Weak or irreproducible immobilization signal	Coupling chemistry involves residues at the interaction site; protein damaged on the surface	Change immobilization chemistry or orientation; use site-specific capture through a tag	Confirm surface activity with a control analyte of known affinity
Kinetic parameters differ between batches	Variable fraction of active protein; different aggregation state	Standardize the protocol; determine the active concentration for each batch	Apply Table 3 to every preparation and compare against a reference

## Drawing conclusions on physiological significance - limitations of using recombinant proteins

### Folding of the recombinant protein may differ from the native state

The folding process of proteins is often described by the so-called folding funnel, which illustrates the theoretical framework of paths and energetic states available to the macromolecule. A protein may reach a partially folded state where the conformation is trapped in a semi-stable well that is characterized by a higher level of the Gibbs free energy (G) than the natively folded protein. A protein in this trapped conformation may be soluble, but inactive or dysfunctional (Nussinov et al., 2026; Giri Rao and Gosavi, 2016). Incorrect disulfide bond formation is a further common source of non-native folding, and it affects in particular those proteins that are secreted in their native context but are expressed in the reducing cytoplasm of *E. coli*.

Folding is therefore a serious problem in protein expression studies, but several strategies have been developed and tested over decades to overcome these problems. When an assay system is at hand to test the function of the protein, one can systematically improve the expression and purification process. These include decreasing the temperature and co-expressing chaperones. Others are targeting the protein to the periplasm, using strains engineered for cytoplasmic disulfide bond formation, and supplementing the culture media with chemicals that support folding (overview in (Beygmoradi et al., 2023; Bhatwa et al., 2021; Hartl et al., 2011; Kim et al., 2013; Lange and Rudolph, 2009; Lilie et al., 1998)). The assay also permits, in the ideal case, the assessment of the relative amount of functional protein with respect to the overall amount of purified protein.

The folding state of a recombinant preparation is an experimentally accessible property, not an assumption. It can and should be verified before any quantitative measurement is interpreted. Far-UV circular dichroism reports on secondary structure content and is the standard first test of whether an expressed protein is folded at all, or whether a mutation has perturbed its conformation (Greenfield, 2006). Near-UV circular dichroism and intrinsic tryptophan fluorescence report on the packing of the tertiary structure and on the burial of aromatic side chains. Thermal denaturation methods return the midpoint of the unfolding transition and its cooperativity. These are differential scanning calorimetry, thermal shift assays with an extrinsic dye, and nano-differential scanning fluorimetry, which follows the intrinsic fluorescence of the protein without any label. A sharp, cooperative transition is itself a signature of a well-packed native fold. Comparing melting temperatures across mutants, or in the presence and absence of a ligand, is one of the most economical experiments available. Size-exclusion chromatography coupled to multi-angle light scattering and analytical ultracentrifugation report on the oligomeric state and on the presence of soluble aggregates that electrophoresis does not detect. Identity and covalent integrity are established by intact-mass measurement and by peptide mapping with liquid chromatography coupled to tandem mass spectrometry. The same analysis localizes posttranslational modifications and quantifies their site occupancy. None of these measurements replaces a functional assay, and none of them alone is sufficient. Taken together, however, they define a minimum set of criteria that a recombinant preparation should satisfy before it is used to answer a biological question. These criteria, the techniques that address them and the interpretation of the corresponding readouts are collected in Table 3.

### Posttranslational modifications

Protein function may critically depend on the correct posttranslational modification. The most common modifications are phosphorylation, methylation, acetylation, SUMOylation, ubiquitination, glycosylation and lipid attachments (Øye et al., 2025; Chen and Kashina, 2021). The suitable choice of the expression system is the first decision to make when planning to obtain a recombinant protein, and it is largely dictated by which of these modifications the target protein requires (see Table 2).

Bacterial expression in *E. coli* is the usual starting point and is routinely used for achieving very high yields of the protein of interest (Structural Genomics Consortium et al., 2008; Rosano and Ceccarelli, 2014; Schütz et al., 2023; de Marco, 2025; Rosano et al., 2019; Aguilar Lucero et al., 2021). For example, small scale expression can reach 10–100 mg/L, in some cases under optimized conditions even 100–800 mg/L. Large scale expression in bioreactors reaches yields of several g/L. The choice of strain is an important and often underexploited variable. BL21(DE3) derivatives carrying additional tRNA genes (Rosetta) compensate for codons that are rare in *E. coli*. Strains with an engineered lactose permease (Tuner) allow graded rather than all-or-none induction, and strains co-expressing cold-adapted chaperonins (ArcticExpress) support folding at low temperature. Strains carrying mutations in the thioredoxin and glutathione reductase pathways (Origami, SHuffle) permit disulfide bond formation in the cytoplasm (Rosano et al., 2019; Aguilar Lucero et al., 2021).

The principal limitation of bacterial hosts is that *E. coli* lacks most of the eukaryotic modification machinery. Some modifications can be reconstituted by co-expressing the required enzyme, for example yeast N-myristoyl-transferase for binding myristic acid to the N-terminal consensus site (Towler et al., 1988; Gordon et al., 1991). Other common lipid modifications in eukaryotic cells are palmitoylation and isoprenylation (Casey, 1994; Wang and Casey, 2016). More than 200 proteins in eukaryotic cells harbour the CaaX sequence at the C-terminus, where a stands for an aliphatic residue and X for any amino acid. This is a consensus sequence for a farnesyl or geranylgeranyl transferase, which modifies the target protein by adding isoprene lipids (Wright and Philips, 2006; Lane and Beese, 2006). One modification, however, cannot be addressed in this way, and it is the most consequential of all.

Glycosylation is quantitatively the most abundant and structurally the most diverse posttranslational modification of eukaryotic proteins, and it is essentially absent from standard bacterial expression systems (Schjoldager et al., 2020). N-glycans are attached to asparagine residues within the Asn-Xaa-Ser/Thr sequon and O-glycans to serine and threonine residues. Both classes influence folding and solubility, thermal and proteolytic stability, subcellular targeting, circulatory half-life and, in many cases, the affinity and the specificity of molecular recognition. A protein whose function depends on its glycans is therefore not simply less active when produced in *E. coli*: it may be inactive, misfolded or prone to aggregation. This is the single most compelling reason to abandon a bacterial host at the planning stage rather than after months of optimization. Bacterial N-glycosylation is not entirely out of reach, since the *Campylobacter jejuni* protein glycosylation locus was functionally transferred into *E. coli*, founding the field of bacterial glycoengineering (Wacker et al., 2002). The glycan structures obtained in this way nevertheless differ from their eukaryotic counterparts, and the approach remains specialized. A further point is relevant to the interpretation of any biophysical experiment. Glycosylation is heterogeneous even in native systems, so a glycoprotein is a population of glycoforms rather than a single molecular species.

Eukaryotic cell culture systems normally contain the required enzymes for modification, at the cost of lower yields, longer timelines and higher expense. Yeast systems perform N-glycosylation but produce high-mannose structures unlike the mammalian ones, unless the strain has been glycoengineered. Insect cell cultures are in routine use for the extraction of higher yields (Mishra, 2020; Huang et al., 2025) and carry out most modifications, although their N-glycans are typically paucimannosidic. Mammalian systems come closest to the native situation. Keeping HEK293 cells in suspension allows up-scaling of protein expression, yielding around 100 mg per Liter of culture (Dash et al., 2023; Subedi et al., 2015). CHO cells are the dominant platform for therapeutic glycoproteins, and have produced the large majority of the mammalian-cell-derived biopharmaceuticals approved over the last two decades (Walsh and Walsh, 2022). For research-scale work, transient transfection of HEK293 or CHO suspension cultures delivers milligram amounts of human-like glycosylated protein within one to two weeks. No stable cell line, and none of the months needed to establish one, is required. It is now the method of choice whenever a limited amount of correctly modified protein is required rapidly (Dash et al., 2023; Subedi et al., 2015).

Cell-free expression occupies a distinct position in this landscape. Transcription and translation are carried out in a lysate rather than in living cells, which removes the constraints imposed by cell viability and by membrane permeability. Yields have improved substantially and reach the gram-per-litre range in optimized *E. coli*-based systems (Silverman et al., 2020). The open reaction format allows the direct addition of chaperones, redox buffers, detergents, nanodiscs, lipids, unnatural amino acids and isotopically labelled precursors. This makes cell-free systems particularly suitable for toxic proteins, for membrane proteins, and for the rapid parallel screening of many sequence variants. Lysates prepared from eukaryotic sources extend the approach to targets requiring more complex processing. The main limitations are the cost per milligram of product and the fact that the modification capacity is that of the lysate rather than that of a living cell.

When a modification cannot be installed biologically, it can be installed chemically. Expressed protein ligation exploits intein-mediated generation of a C-terminal thioester on a recombinantly expressed protein fragment. The fragment is then chemoselectively ligated to a synthetic peptide carrying the desired modification at a defined position (Muir et al., 1998). The product is homogeneous and site-specifically modified, in contrast to the heterogeneous mixtures obtained by enzymatic modification in cells. The approach has been widely used to prepare C-terminally modified ubiquitin conjugates. A particularly informative illustration is the semisynthesis of the R2 subunit of *E. coli* ribonucleotide reductase. Intein chemistry was used there to place unnatural tyrosine analogues at position 356, in order to dissect the long-range proton-coupled electron transfer pathway of the enzyme (Yee et al., 2003). No expression system alone could have delivered that experiment. Genetic code expansion, in which an orthogonal tRNA/synthetase pair directs the incorporation of an unnatural amino acid at a reassigned codon, offers a complementary route inside a living cell (Phromkrasae et al., 2026).

These experimental approaches have been successful in obtaining recombinant proteins with the intended modifications. Verifying the presence, the position and the stoichiometry of the attached residues is a separate problem, and it makes further demands on bioanalytical technique. Intact-mass measurement establishes whether a modification is present and how many copies are carried. Peptide mapping by liquid chromatography coupled to tandem mass spectrometry localizes the modification to a specific residue and quantifies site occupancy, and released-glycan analysis characterizes glycan heterogeneity. These are not routine operations in every biochemistry laboratory, and the need for access to them should be regarded as part of the cost of choosing a modification-dependent target. The characteristics of the main expression systems and the modifications that each of them can deliver are summarized in Table 2.

### Purification and downstream processing

Expression is only half of the problem, and the properties of a recombinant preparation are determined at least as much by the purification route as by the choice of the expression host. A typical scheme has three stages (Oliveira and Domingues, 2018). Capture is most often by affinity chromatography exploiting a genetically encoded tag, intermediate purification by ion exchange or hydrophobic interaction, and final polishing by size-exclusion chromatography. Each of these steps exerts a selection on the population of molecules that survives it, and this selection is not neutral with respect to the questions that will be asked later.

Three points deserve emphasis. First, purity and homogeneity are not the same property. A preparation that migrates as a single band on a Coomassie-stained one-dimensional SDS-PAGE gel may still contain soluble oligomers, partially unfolded species, or a mixture of glycoforms and modification states. What a gel can reveal depends on the size of the modification relative to the size of the protein. A single N-glycan adds roughly 2–3 kDa, a mass increment that is clearly visible for a 20 kDa protein and undetectable for a 150 kDa one. Electrophoretic methods of higher resolution narrow this gap. Two-dimensional electrophoresis separates by isoelectric point as well as by mass, and gels modified to retard a specific modification—as Phos-tag gels do for phosphorylated species, via a phosphate-binding metal complex in the gel matrix—resolve states that a standard gel cannot. None of these methods, however, reports on the oligomeric state in solution. Size-exclusion chromatography with multi-angle light scattering and analytical ultracentrifugation do exactly that. Second, the conditions applied during purification determine whether the protein retains its cofactors, its oligomeric state and its activity. Buffer pH and ionic strength, the presence of reducing agents, chelators and detergents, the number of freeze-thaw cycles and the time elapsed between lysis and measurement all belong to this category. They deserve to be reported with the same care as the expression protocol. Third, the affinity tag that made purification straightforward may interfere with the measurement that follows. It can occlude an interaction surface, alter the oligomeric state, or contribute its own signal to a biophysical experiment. Site-specific proteolytic cleavage at a protease site placed between the tag and the protein of interest removes the tag, at the cost of a further purification step and of some loss of material. The decision to remove the tag or to retain it should be taken explicitly and justified, rather than by default.

Preparative ultracentrifugation deserves separate mention, because it addresses a class of targets that chromatography handles poorly. Density-gradient ultracentrifugation, typically in caesium chloride or iodixanol, separates assembled viral capsids from empty particles and from cellular contaminants. It remains central to the production of adeno-associated virus vectors for gene delivery, where the ratio of full to empty capsids is a critical quality attribute (De-Luca et al., 2024). The same physical principle, applied analytically, gives access to the size distribution and the association state of a protein sample in solution without the need for a chromatographic matrix.

Typical failure modes at the expression and purification stages, together with their probable causes and the corresponding interventions, are collected in Table 4.

### Lack of coenzymes and cofactors

Many enzymatic reactions and functions of proteins depend on the presence of coenzymes (e.g. NAD^+^/NADH; NADPH/NADP^+^; FAD/FADH_2_) and cofactors (e.g. Fe^2+^, Mg^2+^, Mn^2+^), which may be lost during the fractionation and purification process but can be added afterwards. However, more problematic is a coenzyme that needs to be integrated in the protein structure before the folding process during translation is complete. Even more challenging is the case, when no knowledge about necessary coenzymes or cofactors exists, which might require a screening process. Some proteins are intrinsically disordered or contain disordered regions that need the interaction with a stabilizing factor or protein (van der Lee et al., 2014; Skriver et al., 2023). Disordered regions could facilitate aggregation processes leading to insoluble expression products. A common strategy to combat low solubility is to engineer a fusion protein with a solubility tag (Silva et al., 2021; Ki and Pack, 2020; Mamipour et al., 2017). Other tags are chosen to allow detection during the expression and purification steps via anti-tag specific antibodies and to shortcut purification protocols by using affinity chromatography (Structural Genomics Consortium et al., 2008; Rosano and Ceccarelli, 2014). Presence of the fusion part or tag during *in vitro* functional studies could impact the outcome of the experiments. Therefore, site specific proteolytic digestion on protease cleavage sites located between the tag and the protein of interest can remove the tag, which however requires a further purification step.

### Crowded situation in a cell

The cellular environment is characterized by high concentrations of macromolecules, metabolites, lipids, membranes and supramolecular protein complexes. Physiological protein concentrations can sum up to 400 g L^-1^ leading to an occupation of 5%–40% of the interior volume of a cell. Such a situation is termed molecular crowding, or is referred to as the excluded volume effect (Ellis and Minton, 2006; Zhou et al., 2008). Under these conditions proteins may exhibit different properties, for example altered conformational changes or assembly into specific protein complexes. Under test tube conditions, the concentrations of macromolecules are, in the best case, 1–2 orders of magnitude lower leading to a less protective environment. Proteins present under *in vitro* conditions in low concentrations are prone to misfolding and can stick to glass or plastic surfaces by exposing hydrophobic regions. These processes can trigger denaturation and loss of significant protein amounts. This problem can be overcome by working with crowding reagents to simulate the cellular osmotic situation. Crowding reagents should be water soluble, of low viscosity and should not self-aggregate. Widely used compounds are synthetic macromolecules like polyethylene glycol, polyvinyl alcohol, and dextran and Ficoll derivatives (Tokuriki et al., 2004). The conformational changes in Ca^2+^-sensor proteins, for example, can be monitored under the crowding conditions present on biosensor surfaces (Sulmann et al., 2014). Globular proteins may also be used as crowding agents, but only when an interaction with the protein under study can be excluded. Crowding in a cellular environment can lead to severe pathological conditions. Neurodegenerative diseases such as Parkinson’s, Alzheimer’s and Huntington’s are caused by the formation of protein aggregates under cellular crowding conditions.

## Arguments in favor of recombinant proteins

Proteins from native sources (except bacteria or yeast) are in most cases only available in small quantities or would need large amounts of specific organs as a starting material for fractionation. Instead, the production of recombinant proteins for biomedical and biotechnological purposes can relatively easily be scaled up from routine bench application to larger yields. We think that there is a general agreement that purified recombinant proteins for biomedical research or therapy provide useful and effective tools (e.g. human insulin application in diabetes; see below). But what about basic research on general biological processes in combination with physiological recordings? Can we obtain useful information from pure *in vitro* studies to understand a biological phenomenon on the cellular, physiological level? We have collected some main applications of recombinant proteins in basic research and discuss their advantages.

### Protein function at the molecular level

In case the general function of a protein is known or suggested based on educated guess, the next step of biochemical analysis often requires extensive sets of experiments. Classical enzyme kinetics for the determination of catalytic parameters *K_m_*, *V_max_*, and the impact of modulatory or inhibitory factors need rigorously clean samples. Very often, enzyme preparations from native sources are not available at high yields, making the use of recombinant proteins necessary. A further clear advantage comes with the generation of mutants. Site-directed mutagenesis is a routine technique in many laboratories worldwide. It allows critical amino acid positions to be inspected for their role in enzyme catalysis and in protein function. It also serves to probe the binding and the effect of modulatory factors, and to investigate disease-related mutations. The list is not complete and can be extended, but it shows already the wide range of applications of recombinant proteins.

Two parameters summarize the behaviour of a Michaelis-Menten enzyme. The Michaelis constant *K_m_* is the substrate concentration at which the reaction proceeds at half its maximal rate. It provides an operational measure of the affinity of the enzyme for its substrate: a low *K_m_* indicates that saturation is reached at low substrate concentration. The maximal velocity *V_max_* is the rate approached when the enzyme is saturated, and it depends on the amount of enzyme present. Dividing *V_max_* by the total enzyme concentration gives the turnover number *k*_cat_, the number of substrate molecules converted per active site per unit time. The ratio *k*_cat_/*K*_m_, often called the specificity constant, describes catalytic efficiency at low substrate concentration. It is the parameter of choice when comparing an enzyme with its mutants or with alternative substrates. These parameters are not equally sensitive to the quality of the preparation, and the distinction matters in practice. *K*_m_ is obtained from the dependence of the initial rate on substrate concentration and does not require the enzyme concentration to be known. For a given protein in a given buffer it should therefore be comparable across preparations and across laboratories, whether or not part of the material is inactive. *V_max_* and *k*_cat_ behave differently. Since *k*_cat_ is *V_max_* divided by the total enzyme concentration, any inactive fraction—misfolded, aggregated, or lacking a cofactor—inflates the denominator and depresses the apparent *k*_cat_ in direct proportion. Determining the concentration of active enzyme, rather than of total protein, is therefore a prerequisite for reporting *k*_cat_, and the ratio *k*_cat_/*K*_m_ inherits the same error. The point is not marginal when mutants are compared. A substitution that partly destabilizes the fold reduces the fraction of active molecules and therefore lowers the apparent *k*_cat_, even though the intrinsic catalytic rate of the correctly folded molecules is unchanged. *K*_m_, measured on the same sample, is unaffected. A fall in apparent *k*_cat_ accompanied by an unchanged *K*_m_ is therefore a signature of an inactive fraction rather than of a genuine catalytic defect.

### Interaction studies in a well-defined setting

A critical aspect of protein function is interactions with other macromolecules (proteins, DNA, RNA, and oligosaccharides), metabolites, ligands or associations with biological membranes. Purification of recombinant proteins can provide sufficient material for extensive direct interaction studies. Several methods are directed to establish in principle whether an interaction of two proteins exists. For example, yeast two-hybrid assays allow singling out interaction partners in a large set of expressed proteins that might even cover a whole proteome (Fields and Sternglanz, 1994; Johnsson and Varshavsky, 1994; Brückner et al., 2009). The proximity-ligation assay works with fusion constructs where the protein of interest is combined with a biotin ligase. Any binding protein in direct vicinity of the ligation-fusion construct can be biotinylated within a range of 10–15 nm and can be identified using a combination of biotin-streptavidin detection and mass spectrometry. Proteins in these assays are expressed in recombinant form, but act in a cellular environment that might provide a more physiological setting (Lundberg et al., 2011; Li et al., 2025). While not absolutely required, recombinant proteins are practically essential for reliable, reproducible and interpretable protein array interaction studies. Such studies are also useful to identify relevant interactions within biochemical pathways. Methodological descriptions and discussions about these and other cellular based experimental approaches can be found elsewhere in more detail and are not the focus of this tutorial guide. Instead, we ask other fundamental questions related to the phenomenon of protein-protein interaction: what is the affinity of the interaction process? What are the kinetic parameters that determine the binding step? What is the thermodynamic driving force? Finally, do these experimental data obtained with recombinant proteins have a physiological meaning? Do they match physiological recordings or observations? We will discuss some widely used techniques to answer these questions.

#### Fluorescence based methods

Fluorescence-based methods exploit a property that most proteins already possess. Three of the twenty canonical amino acids are intrinsically fluorescent: phenylalanine, tyrosine and tryptophan. Their quantum yields differ by more than an order of magnitude, and tryptophan is by far the brightest of the three. Energy transfer from tyrosine to tryptophan and the low absorbance of phenylalanine add to this. On excitation near 280–295 nm, the emission of a typical protein is therefore dominated by its tryptophan residues. What makes tryptophan a useful reporter is not only its brightness but the strong dependence of its emission on the local environment. A tryptophan buried in the hydrophobic core of a protein emits with high intensity and a maximum near 330 nm, whereas the same residue exposed to water emits more weakly and with a maximum shifted towards 350 nm. Several kinds of event change the solvent exposure or the polarity around a tryptophan. Examples are a conformational transition, the binding of a ligand or of a partner protein, the approach of a membrane surface, and the loss of the native fold. Each produces a measurable change in emission intensity, in the position of the emission maximum, or in both. This is the physical basis of the experiments described below, and it requires no chemical modification of the protein. The main limitation is the converse of the main advantage. The signal is an average over all tryptophan residues present, so proteins containing several of them give a response that is hard to assign to a specific site. Site-directed mutagenesis, used to remove or introduce individual residues, resolves this.

Proteins undergo conformational changes, when they bind ligands, ions, biological membranes or interact with target proteins. These conformational transitions are often important trigger steps in signaling pathways making them preferred study objects. Intrinsic tryptophan residues are therefore widely used for monitoring conformational changes (Figure 3). The binding of Ca^2+^ to calcium-binding proteins is a prominent example where the changes in tryptophan fluorescence emission are employed to investigate Ca^2+^-dependent conformational changes. In combination with site-directed mutagenesis, a deeper investigation of protein structure-function relationships can be carried out (Peshenko and Dizhoor, 2006; Peshenko and Dizhoor, 2007; Marino et al., 2015; Vocke et al., 2017). Other examples are the α-subunits of heterotrimeric G proteins. These harbour a tryptophan at a conserved position, whose fluorescence emission intensity changes when the α-subunit transits to the active GTP-bound state (Faurobert et al., 1993; Preininger et al., 2008; Görtemaker et al., 2022). Purified recombinant proteins are used in these studies, either to prove the presence of a somehow active protein (transitions to the active state) or to determine the dissociation constant of a ligand-protein interaction.

**FIGURE 3:**
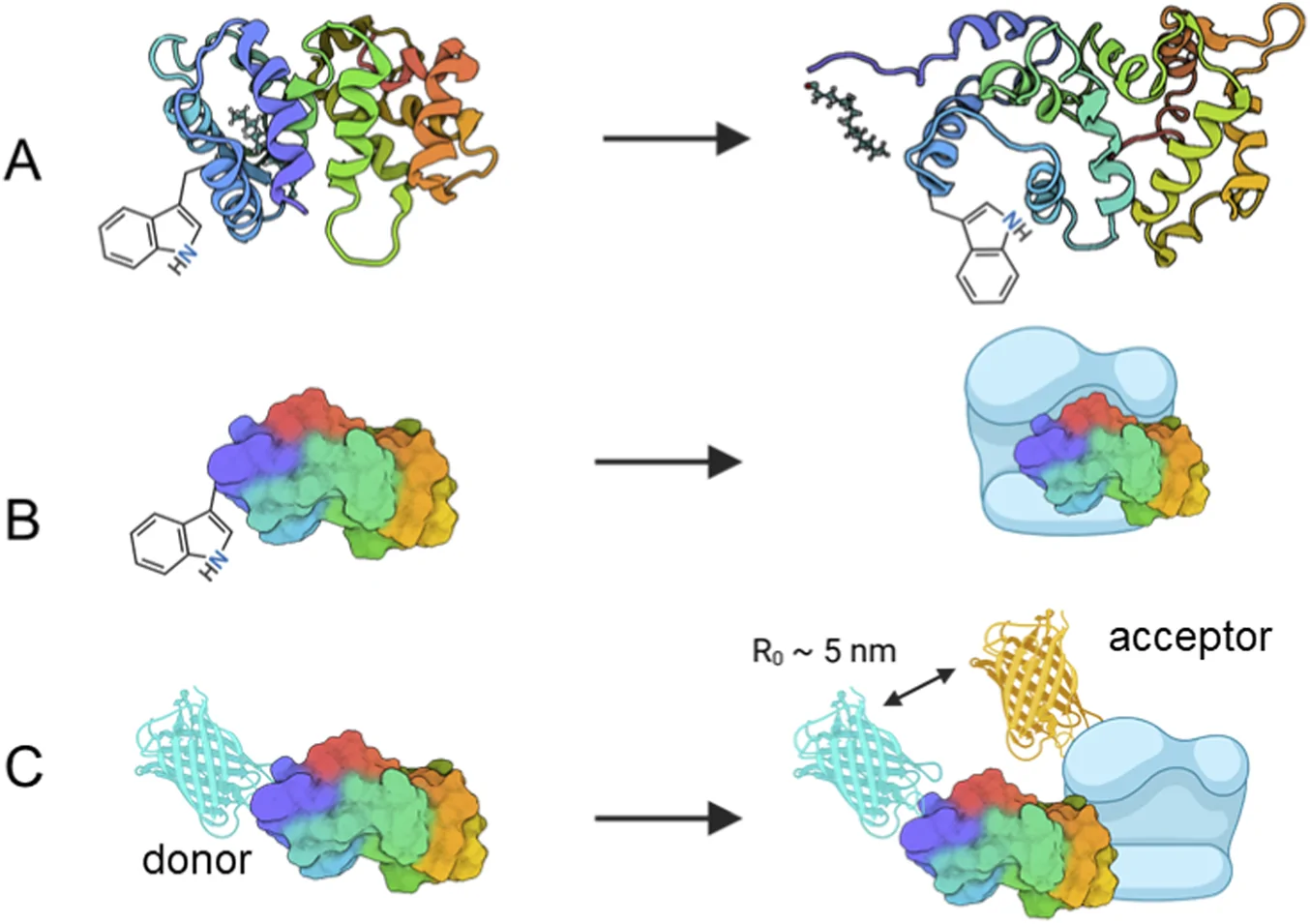
Application of fluorescence spectroscopy to study molecular properties of proteins. Intrinsic tryptophan fluorescence in a protein serves several distinct aims. Conformational changes monitored by an intrinsic tryptophan are detected as a shift in the tryptophan fluorescence emission **(A)**. Protein-protein or protein-ligand interactions are followed by the change in the emission signal upon complex formation using tryptophan fluorescence emission as well **(B)**. An experimental set up for FRET analysis is illustrated in **(C)**. Two interacting proteins are labelled with different dyes representing a matching FRET pair, for example Cyan-fluorescent protein as donor and yellow fluorescent protein as acceptor. The Förster radius of R_0_ ∼ 5 nm for optimal resonance transfer is indicated.

*Fluorescence (or Förster) resonance energy transfer (FRET)* — Most applications of FRET are currently in cell culture systems. Most of these studies do not determine quantitative parameters of the interaction process (Jares-Erijman and Jovin, 2003; Shaner et al., 2005; Bajar et al., 2016). An imaging-based FRET assay was developed and optimized to determine dissociation constants for the interacting protein pair of interest in a cell-based environment (de Las Heras-Martínez et al., 2018). The approach requires the determination of several additional parameters including an exact value of the FRET efficiency. Alternatively, titration measurements in solution using donor-acceptor FRET pairs were developed (Liao et al., 2015). Keeping one binding partner constant by varying the concentration of the other does not differ from biochemical titration series. Although the approach seems straightforward, it requires the expression and purification of protein pairs tagged with matching donor-acceptor fluorescence compounds. It also requires a set of controls that correct the direct emission of donor and acceptor. The production of recombinant proteins is therefore necessary.

#### Surface plasmon resonance and other sensor-based techniques

Studying the kinetics of protein-protein interactions—how fast they bind and unbind—helps us to understand the dynamics and timing of biological processes, not just how strongly proteins stick together (affinity). Fundamental parameters describing an interaction process are the association (*k* ^*on*^) and dissociation (*k* ^*off*^) rate constants yielding the equilibrium or dissociation constant (K_d_). Surface plasmon resonance (SPR) is widely used for interaction studies of biomolecules and provides kinetic constants of complex formation. It also yields biologically relevant information under conditions where equilibrium is not reached (Schuck, 1997; Karlsson et al., 2006; Zhao et al., 2017). SPR is an optical phenomenon that is observed at the interface of two media of different refractive index (a thin metal film and a compartment containing an aqueous buffer). The critical condition of the detection principle is realized when light hits a sensor chip with thin metal film under total internal reflection, for which a specific prism is used. An evanescent electric field extends into the medium of lower refractive index. Under specific conditions the incident light energy is converted into electron density waves in the metal film, which leads to a resonance phenomenon at the reflecting surface. Binding events on the surface of the chip are sensed as a consequence of the resonance phenomenon and can be recorded as a time-dependent change in so-called resonance units. The recording includes an association phase and a dissociation phase, from which kinetic parameters can be obtained by suitable curve fitting. A typical set of SPR recordings and the evaluation of the association and dissociation phases of the binding curve is illustrated in Figure 4. The technique is widely applied for the characterization of antigen-antibody interactions, either for basic research or biomedical diagnostics (Alfthan, 1998; Huber et al., 1999). In addition, other kinds of biomolecular interactions have been investigated by SPR including cytoplasmic and membrane proteins, peptides, nucleic acids, oligosaccharides and bilayer membranes (Rich and Myszka, 2007; Dell’Orco and Koch, 2011). For recordings in a defined setting, purified proteins are used. These are (in most cases) recombinant proteins that have been characterized by standard biochemical methods, which could include functional assays if available or possible. However, the technique has several drawbacks adding to the general critique of using recombinant proteins. The immobilization step could create obstacles such as that the critical protein surface is not accessible or that the immobilized protein is damaged by the chemical binding procedure. Furthermore, conformational changes of an immobilized protein essential for the binding process could be hampered. However, any of these pitfalls can be addressed by modifying the experimental setup (Myszka, 1999). The immobilization strategy, chemistry or geometry can be changed, for example by adding tags that permit a site-specific immobilization which does not hinder the ligand surface area that is active in the recognition process. More recently, SPR was applied to monitor conformational changes in proteins, by correlating changes in the hydration shell of the protein to the dielectric milieu of the sensor chip surface (Sulmann et al., 2014; Dell’Orco et al., 2010a; Dell’Orco et al., 2012; Dell’Orco and Koch, 2016). The approach shows high sensitivity and permits detailed comparisons among homologous proteins of the same family.

**FIGURE 4:**
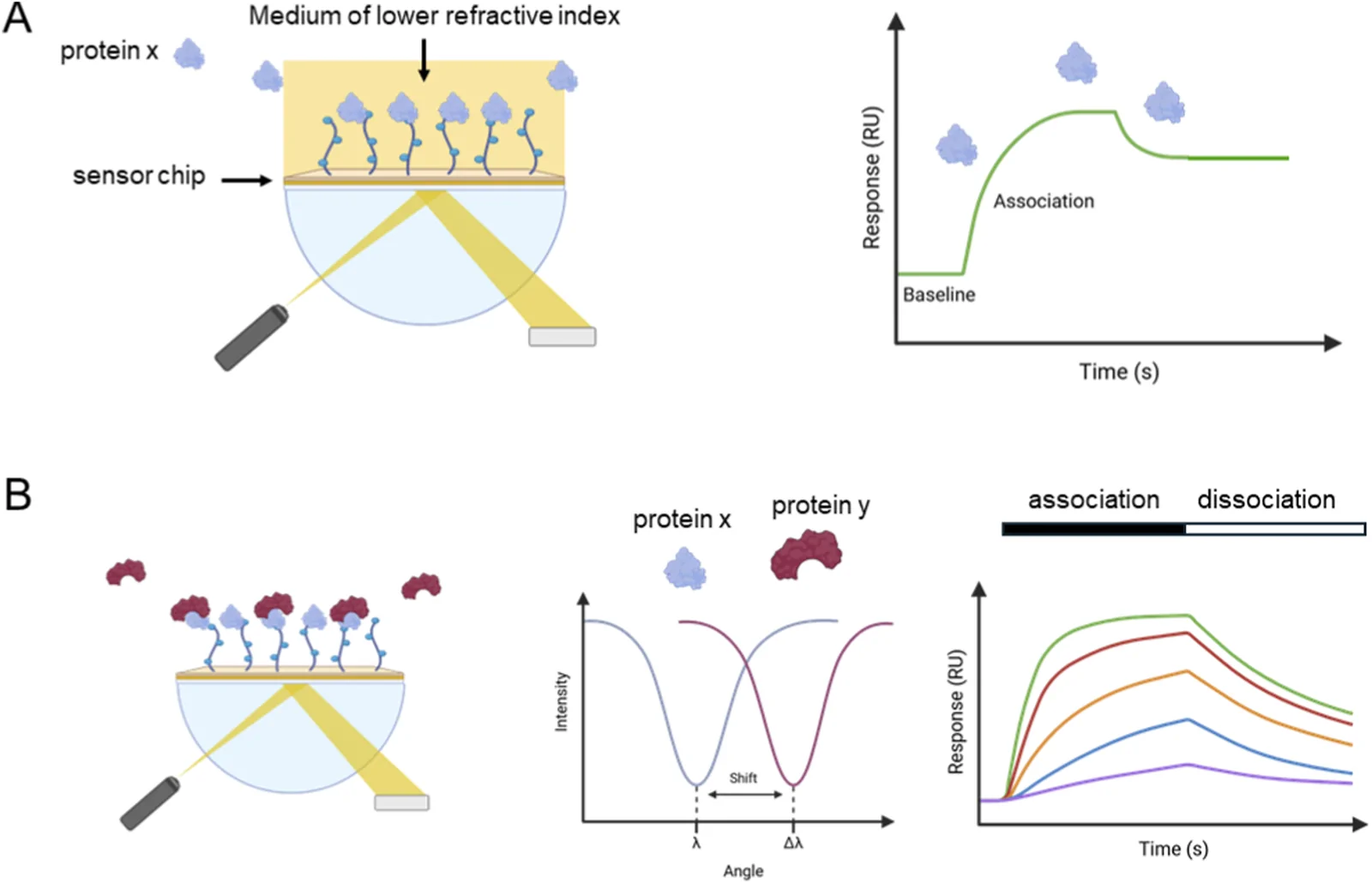
Experimental setup of SPR measurements. **(A)** Left part: a light source emits monochromatic light that traverses through a prism at a certain angle and that is totally internally reflected at an interface of two media. A thin metal layer is at the boundary between the prism and a medium of lower refractive index (e.g. aqueous buffer). The protein of interest (protein x) is immobilized on an activated carboxylated layer by covalent attachment leading to a positive response signal (right part). Flushing the surface with buffer will remove loosely associated protein x, but a significant part stays on the sensor chip indicated by the straight line. **(B)** Protein y is injected onto the flow system over the protein x coated sensor chip (left part). A binding event causes a shift in the minimum of reflected light intensity (middle part) that can be recorded in a time-dependent manner, yielding sensorgrams with amplitudes increasing with the analyte (protein y) concentration (right part).

Backscattering interferometry (BSI) is also based on refractive index changes. The optical setup consists of a long-wavelength laser beam, a microfluidic system for sample application and a detection system such as a CCD camera (Baksh et al., 2011). It has the advantage of detecting molecular interactions in a label-free approach. The method does not depend on immobilization of one interaction partner allowing analysis of binding events under physiologically relevant conditions with high sensitivity and in small volumes (Baksh et al., 2011; Kammer et al., 2018; Sulmann et al., 2017).

Bio-layer interferometry (BLI) and Grating-Coupled Interferometry (GCI) are two label-free optical biosensor methods that, like SPR, detect molecular binding events through changes in local refractive index. They differ substantially from SPR, and from each other, in their physical implementation and practical scope. In BLI, a fiber-optic probe bearing the immobilized ligand is dipped directly into the analyte solution. Binding events alter the optical thickness of the biolayer on the probe tip, which is detected as a shift in the interference pattern of reflected white light. This tip-based, flow-free format simplifies the experimental setup, reduces sample volumes, and is well suited for parallel, high-throughput kinetic measurements (Bates et al., 2025). GCI, by contrast, relies on a planar waveguide in which light is guided along the sensor surface after being deflected into it by a diffraction grating. The resulting evanescent field detects mass accumulation at the sensor surface with exceptional sensitivity. It covers an unusually wide affinity range and enables reliable kinetic measurements even for very small analytes, including low-molecular-weight compounds and metal ions (Jankovics et al., 2020). Both techniques require immobilization of one binding partner, a limitation shared with SPR. The choice among the three methods is therefore often dictated by the molecular weight of the analyte, the required throughput and sample availability.

#### Calorimetry-based methods

A method complementary to SPR is isothermal titration calorimetry (ITC) (Brautigam et al., 2016; Falconer et al., 2021). The method does not need a label like in fluorescence studies or the immobilization of one binding partner on solid support as in SPR. Instead, the method can be applied in solution to investigate a wide range of interaction processes such as protein-protein, protein-ligand, protein-ions, protein-lipids, protein-carbohydrate and protein-nucleic acids (Falconer and Collins, 2011; Marino et al., 2017; Abbas et al., 2019; Abbas and Koch, 2020; Salim and Feig, 2009). Since nearly all binding events release or consume heat, a sensitive and precise recording of heat changes is used for thermodynamic parameters. The Gibbs free energy of the binding process ΔG_a_ relates to the association constant (K_a_), enthalpy (ΔH) and entropy (ΔS) as follows:

$$\Delta G_{a}=-\text{RT}\;\text{ln}\left(K_{a}\right)=\Delta H_{a}-T\Delta S_{a}$$

R is the gas constant and T the absolute temperature in K.

One binding partner in a typical calorimetric measurement is provided in the measuring cell. The other binding partner can be injected dropwise into the measuring compartment under constant stirring. A titration series can result in release or consumption of heat producing exothermic or endothermic heat pulses. Evaluation of heat pulses gives values of K_a_ and ΔH_a_ used for further calculations based on the relationships shown above. ITC is a method to provide thermodynamic parameters in addition to binding constants and the experimental approach requires purified proteins in higher amounts than in fluorescence studies. Yielding thermodynamic parameters of an interaction process by ITC adds to a deeper understanding of molecular processes, which cannot be obtained from cellular recordings or the analysis of whole organs or organisms.

A technique complementary to ITC is Differential Scanning Calorimetry (DSC). While ITC measures heat exchanged during a binding event at constant temperature, DSC monitors changes in the heat capacity of a protein solution as the temperature is gradually increased. The measurement captures the thermodynamic signature of the unfolding transition. The key output is the melting temperature (T_m_), i.e., the temperature at which half of the protein population is unfolded, together with the enthalpy and heat capacity change associated with the transition. DSC is therefore primarily a tool for assessing protein stability rather than binding affinity. It is particularly valuable for verifying that a recombinant protein is correctly folded, for comparing the stability of wild-type and mutant variants, and for evaluating the effect of ligand binding on thermal stability (Johnson, 2013).

The complementary use of ITC, DSC and SPR is exemplified by studies on calmodulin (CaM), a ubiquitous calcium sensor protein that regulates over 300 molecular targets. The combination of calorimetric and biosensor-based approaches, applied to recombinant wild-type and mutant CaM proteins expressed in *E. coli*, allowed a thorough thermodynamic and kinetic characterization of CaM recognition of specific targets, including ryanodine receptors (Dal Cortivo et al., 2025; Dal Cortivo et al., 2023; Dal Cortivo et al., 2022). Point mutations associated with congenital arrhythmias were shown to alter binding affinity, dissociation kinetics and thermal stability, and thereby to perturb the selectivity of molecular recognition.

### Determination of three-dimensional structures of proteins

The structural approach to enzyme mechanism begins with a protein purified from a native source and requiring no recombinant technology at all. In 1965 the structure of hen egg-white lysozyme was solved at 2 Å resolution by David Chilton Phillips and co-workers, the first enzyme structure ever determined (Blake et al., 1965). A bound substrate analogue could be visualized within the cleft of the molecule, so the structure did more than describe a fold. It allowed a chemical mechanism to be proposed directly from atomic coordinates, and in doing so established structural enzymology as a discipline. The rhodopsin and β2-adrenergic receptor structures discussed below belong to the same intellectual lineage, extended to targets that could not be obtained in the quantities lysozyme afforded.

The structure-function relation of macromolecules is one of the fundamental concepts of modern biology and the determination of the three-dimensional structure of proteins and protein complexes is an ongoing effort. NMR spectroscopy, X-ray crystallography and single-particle cryogenic electron microscopy are the major techniques used to obtain structural information on proteins (Vedadi et al., 2010). The last of these has changed the balance among them. Direct electron detectors and improved image-processing algorithms lifted cryo-EM from a low-resolution technique to one that routinely reaches atomic or near-atomic resolution. Three of its properties are decisive for the subject of this review. It requires far less material than crystallography, typically micrograms rather than milligrams. It also requires no crystals, which removes the step at which projects on large complexes and on membrane proteins most often fail. It resolves conformational heterogeneity rather than averaging it away, since multiple states present in the same sample can be classified and reconstructed separately. This brings structural work closer to the dynamic picture that *in vitro* measurements otherwise provide only indirectly (Egelman, 2016). Structural techniques differ in their special applications and potential (size and possible resolution in Å), but they all require enough purified protein. While native protein sources can be used only in very limited cases, the vast majority of proteins need to be produced in recombinant form. A notable exception is rhodopsin. Its three-dimensional structure was solved by X-ray crystallography using protein purified from bovine rod outer segments, and it was the first high-resolution structure of a G protein-coupled receptor (Palczewski et al., 2000). However, such cases are rare, and the challenge of producing sufficient quantities of correctly folded recombinant protein—particularly for membrane proteins—long represented the main bottleneck in structural biology. The crystal structure of the β2-adrenergic receptor in complex with its cognate Gs protein, determined by Rasmussen, Kobilka and colleagues, is widely regarded as the “holy grail” of GPCR structural biology (Rasmussen et al., 2011). It required decades of methodological refinement in recombinant protein production and crystallization, and it ultimately contributed to the award of the 2012 Nobel Prize in Chemistry.

More recently, the AlphaFold algorithm (Jumper et al., 2021) revolutionized structural biology by predicting the three-dimensional structure of proteins from their amino acid sequence alone. This works even for targets that have never been solved experimentally. The field has moved rapidly since. RoseTTAFold demonstrated that comparable accuracy could be reached with a three-track architecture. Protein language models such as ESMFold made prediction fast enough for metagenomic scale, at some cost in accuracy (Lin et al., 2023). The scope has also broadened from single chains to biomolecular complexes. AlphaFold3 predicts the structures of assemblies containing proteins, nucleic acids, small-molecule ligands, ions and modified residues within a single framework (Abramson et al., 2024). Its accuracy exceeds that of specialized docking tools for protein-ligand interactions, and open implementations of the same class of models are now available. Two qualifications are essential for the argument of this review. First, prediction accuracy is not uniform. It degrades for orphan sequences with few homologues, for intrinsically disordered regions, for the effects of point mutations, and for conformational states other than the one most represented in the training data. Second, and more fundamentally, a prediction is a hypothesis about a structure and not a measurement of it. It carries no information on affinity, on kinetics, on thermodynamics, on the population of alternative states, or on the response to a ligand. Every one of these quantities still requires purified protein. Structure prediction has therefore not reduced the demand for recombinant proteins; by generating testable structural hypotheses at a rate no experimental pipeline can match, it has increased it. Predicted structures require experimental validation, for which recombinant proteins remain indispensable. More broadly, the fundamental challenge of relating static structural snapshots to dynamic biological phenomena persists. It is often argued that the conditions of a living cell are far removed from those under which structural and biophysical data are collected. The cell has high macromolecular concentrations, of the order of 400 g × L^-1^, and a complex chemical milieu. This objection is well founded for dilute solution measurements, but it does not apply uniformly, and the crystal is in fact the wrong target for it. Protein crystals are not densely packed arrays of molecules. Solvent occupies between roughly 27% and 65% of the crystal volume, most commonly close to 43% (Matthews, 1968). The protein concentration within a crystal therefore amounts to several hundred grams per litre. In this specific respect a crystal is closer to the interior of a cell than any other *in vitro* system, and considerably closer than the micromolar solutions used in most spectroscopic and calorimetric experiments. What distinguishes the crystal from the cytoplasm is not the degree of crowding. It is the identity of the crowders, the restriction of large-scale motions imposed by the lattice, and the absence of the chemical complexity of the cellular milieu. Stating the objection precisely, rather than in absolute terms, is more useful to the reader who has to decide which technique to apply to which question.

### Persisting objections about the interpretation of *in vitro* studies

A more general critical question about *in vitro* experiments using recombinant proteins remains: do experimental data obtained by fluorescence-based methods, SPR or ITC have a physiological meaning? One might further argue that the modification of binding partners could affect the binding process or even prevent the interaction. What insights into biological processes can be gained from a crystal structure of a protein, which simply represents a snapshot of a highly flexible macromolecule? In summary, the central point of criticism remains, whether the *in vitro* data match the physiology or biological responses of the investigated system. Do recombinant proteins exhibit the same properties *in vitro* as they do in a living cell or organism?

These questions are legitimate, and they have answers. The data obtained *in vitro* describe a defined molecular system under stated conditions, and they are valid within those boundaries. Whether they also describe the behaviour of the same molecule inside a cell is not a matter of principle but an empirical question, to be settled by testing the predictions that the *in vitro* data generate. The remainder of this article is concerned with cases in which this test has been carried out.

## Recombinant proteins: a natural bridge between *in silico*, *in vitro* and *in vivo* research

Beyond the fact that recombinant proteins have pros and cons—as discussed in the previous paragraphs—their use in biological, biomedical, and biotechnological research has increased dramatically in recent decades. This is because a recombinant protein represents an element that is relatively easy to control and characterize in biochemical detail. The information obtained from such detailed characterization can be extremely useful for very different research approaches. Structural and kinetic information can be used to parameterize and properly set up molecular simulations and computational experiments. These aim at determining, *in silico*, properties of individual molecules, molecular complexes, and the entire biological network to which they belong. Such properties are often difficult to obtain experimentally (Dell’Orco, 2009). Moreover, the reconstitution of even complex systems *in vitro*, starting from the available molecular components—including recombinant proteins—can provide insights that are relevant for their *in vivo* counterparts. In the following sections, we will examine some examples of applications of recombinant protein technology in this regard.

### Biomedical and biotechnological applications of recombinant proteins

Recombinant proteins have become a hallmark of biomedical and biotechnological applications. The most prominent example of the biomedical impact of recombinant protein technology is the production of human insulin for the treatment of diabetes mellitus. Insulin was first isolated in 1921 and initially purified from bovine and porcine pancreatic tissue for clinical use. That process was inherently limited in yield, and it was associated with immunogenic complications arising from minor sequence differences between animal and human insulin (Skyler, 1982). The advent of recombinant DNA technology made it possible to produce human insulin of defined sequence in *E. coli* at virtually unlimited scale, representing the first recombinant protein to reach clinical application and marking a landmark in the history of biotechnology (Skyler, 1982; Olorunsola et al., 2021). The recombinant human insulin produced in this way was shown to be indistinguishable from the native human hormone in chemical composition, physical properties and biological activity. Its use substantially reduced the immunogenicity problems associated with animal-derived preparations (Skyler, 1982). Today, recombinant insulin and its engineered analogues remain the cornerstone of diabetes management worldwide (Olorunsola et al., 2021). The analogues differ from the native sequence by targeted amino acid substitutions that modify pharmacokinetic properties.

Furthermore, the understanding of human disorders originating from protein misfolding or amyloid-like plaque formation (eg. prion diseases, Alzheimer’s, Parkinson’s and related neurodegenerative disorders) relied critically on the use of recombinant proteins and *in vitro* studies of aggregation phenomena (Chiti and Dobson, 2017). The study of refolding processes, either supported by chaperones or by other *in vitro* supplements is connected to biomedical research areas and might open routes for therapeutic interventions as well.

Finally, proteins are increasingly viewed as agents for therapeutic purposes aiming at more complex interventions including reprogramming antibodies, creating nanobodies, fusion proteins, and self-assembling biomaterials. Therapeutic applications can be found in oncology, inflammation, metabolic disorders and others (López-Laguna et al., 2026). In each of these cases the requirements for a sound application are the same: stringent tests concerning folding and functional *in vitro* tests, carried out before the protein is used.

### Investigation of protein function in model and non-model organisms

Genetically modified animals are widely used to investigate the consequences of disease-causing mutations or to investigate the function of proteins that are poorly or not understood at all. Common vertebrate animals are mice and zebrafish, depending on the research question. The clear advantage of the experiments with transgenic animals is the “*in vivo*” situation allowing conclusions about function and (disease related) dysfunction. There are however, several drawbacks. For example, the genetically modified animal might not show an apparent phenotype or may not be suitable in general, because its specific tissues or physiological processes differ too much from the human counterpart. The phenotype or genetic background of an animal (e.g. mice) can change over generations (genetic drift), making comparisons with previously published work difficult. Ethical aspects raise concerns about the general use of animals in research and researchers face increasing opposition by animal rights activists. As a consequence, the administrative and bureaucratic burden of applying for a permit to work with animals in research has increased in recent years. Under these circumstances, cell culture methods in combination with recombinant proteins, either expressed in cell culture or in purified form are more and more requested by officials, grant giving institutions and their referees.

Organoids represent an intermediate level of complexity between an animal and a pure *in vitro* test tube approach. They might not be suitable for every research question, and their successful establishment requires significant time and experimental effort. However, multidimensional cell culture systems, such as organoids and tissue-on-chip platforms, are proving invaluable for modeling human development and disease in a more physiologically relevant context. For example, 3D retinal organoid cultures derived from human pluripotent stem cells have been instrumental in studying retinal development, degeneration, and potential therapies for inherited retinal diseases (Kurzawa-Akanbi et al., 2024). Additionally, retina explants obtained from animals destined for the food industry, such as pigs, can be maintained in culture for several days. They offer a valuable *ex vivo* model for testing drug effects, for studying retinal physiology, and for conducting repeated experiments without the need for live animal testing (Weller et al., 2024). In these systems, recombinant proteins such as growth factors, signalling molecules or disease-relevant proteins can be crucial. They serve to modulate cellular behaviour, to mimic pathological conditions, and to evaluate the effects of targeted therapies in a controlled and reproducible way (Asteriti et al., 2023; Dal Cortivo et al., 2026).

The term non-model organism is used here for species that have not been established as standard laboratory systems. Such species lack the infrastructure that defines mice, zebrafish, *Drosophila*, *C. elegans* and *Arabidopsis*. That infrastructure comprises inbred lines, annotated reference genomes, transgenic and knockout technology, antibody and reagent collections, and large communities of users. Two developments have brought such species into the focus of biological research. Genome and transcriptome sequencing has become inexpensive enough that the absence of a reference genome is no longer a barrier to molecular work. It has also become clear that some questions cannot be asked in the standard models, because the phenomenon of interest is absent from them. The choice of a non-model organism is therefore usually dictated by the phenomenon rather than by convenience. It carries a characteristic cost: the animals are hard or impossible to obtain in numbers, genetic manipulation is often unavailable, and native material for biochemistry is scarce. This combination is precisely the one in which recombinant proteins cease to be one option among several and become the only practicable route to molecular-level information. Two species recently brought into experimental use illustrate this. The African spiny mouse (*Acomys*) regenerates skin and other tissues without scarring, a capacity absent from laboratory mice (Seifert et al., 2012). The sea urchin is a long-standing system in developmental biology, and its sequenced genome revealed an unexpectedly complex immune gene repertoire (Hibino et al., 2006). A third example is developed in detail below.

Restricted availability of animals for research requires more *in vitro* experiments in combination with only a limited number of *in vivo* or *ex vivo* experiments. A good example is the research on magnetoreception of migratory songbirds (Hore and Mouritsen, 2016). The geomagnetic field is very likely detected by cryptochrome proteins expressed in the retina of navigating birds. However, research on magnetoreception in birds focused for decades on behavioural studies. These continue today, complemented by neuro-anatomical and physiological studies of bird tissue, mainly whole brain and retina. The scarce availability of birds for research (see above) nearly excludes biochemical experiments with native proteins extracted from the retina. Furthermore, transgenic songbirds have not been created so far, although first attempts for using adeno-associated virus (AAV)-mediated (as in Seth et al., 2025) genetic manipulation of avian photoreceptor cells were recently published (Seth et al., 2025). Thus, cellular and molecular research questions were addressed by employing recombinant proteins shedding light on the key events in magnetic sense signaling. Cryptochrome 4a (Cry4a) from European robin showed photochemical properties in agreement with the molecular properties required for a magnetoceptor protein (Xu et al., 2021). First candidates of downstream signaling of Cry4a were investigated by direct protein-protein interaction studies revealing the α-subunit of the cone specific heterotrimeric G protein Gtα as a promising candidate (Görtemaker et al., 2022).

### Computational modeling of biological responses of protein networks

A biological network based, for example on enzyme activities, protein-protein interactions, changes in second messenger concentrations, ion-channel regulation, and feedback regulation can be modelled. The parameters characterizing these processes must first be obtained by biochemical and physiological investigations.

Kinetic and thermodynamic parameters obtained from *in vitro* experiments using recombinant proteins can serve as the quantitative foundation for computational models of biological networks. Such models enable *in silico* predictions that go well beyond what is experimentally accessible (see Figure 5 to illustrate the integration of biophysical and computational investigations). A prominent example is the vertebrate phototransduction cascade, a G protein-coupled signalling network. Its biochemical complexity has been progressively captured by bottom-up deterministic and stochastic mathematical models, built using parameters derived mostly from recombinant protein studies (Dell’Orco et al., 2009; Invergo et al., 2014; Beelen et al., 2021). These models reproduce the electrophysiological responses of rod photoreceptors across a wide range of light stimuli. Crucially, they also allow quantitative predictions of the effects of point mutations and of changes in the expression levels of individual cascade components. These are predictions that were validated against experimental data from genetically modified animals. In a different but conceptually related application, kinetic parameters obtained by SPR measurements of recombinant proteins interacting with synthetic nanoparticles were used to model the time evolution of the protein corona. The corona is the dynamic layer of plasma proteins that forms on nanoparticle surfaces upon contact with biological fluids. The model correctly predicted the temporal shift in corona composition from an early albumin-dominated state to a late equilibrium dominated by high-affinity apolipoproteins, a result subsequently confirmed experimentally (Dell’Orco et al., 2010b).

**FIGURE 5:**
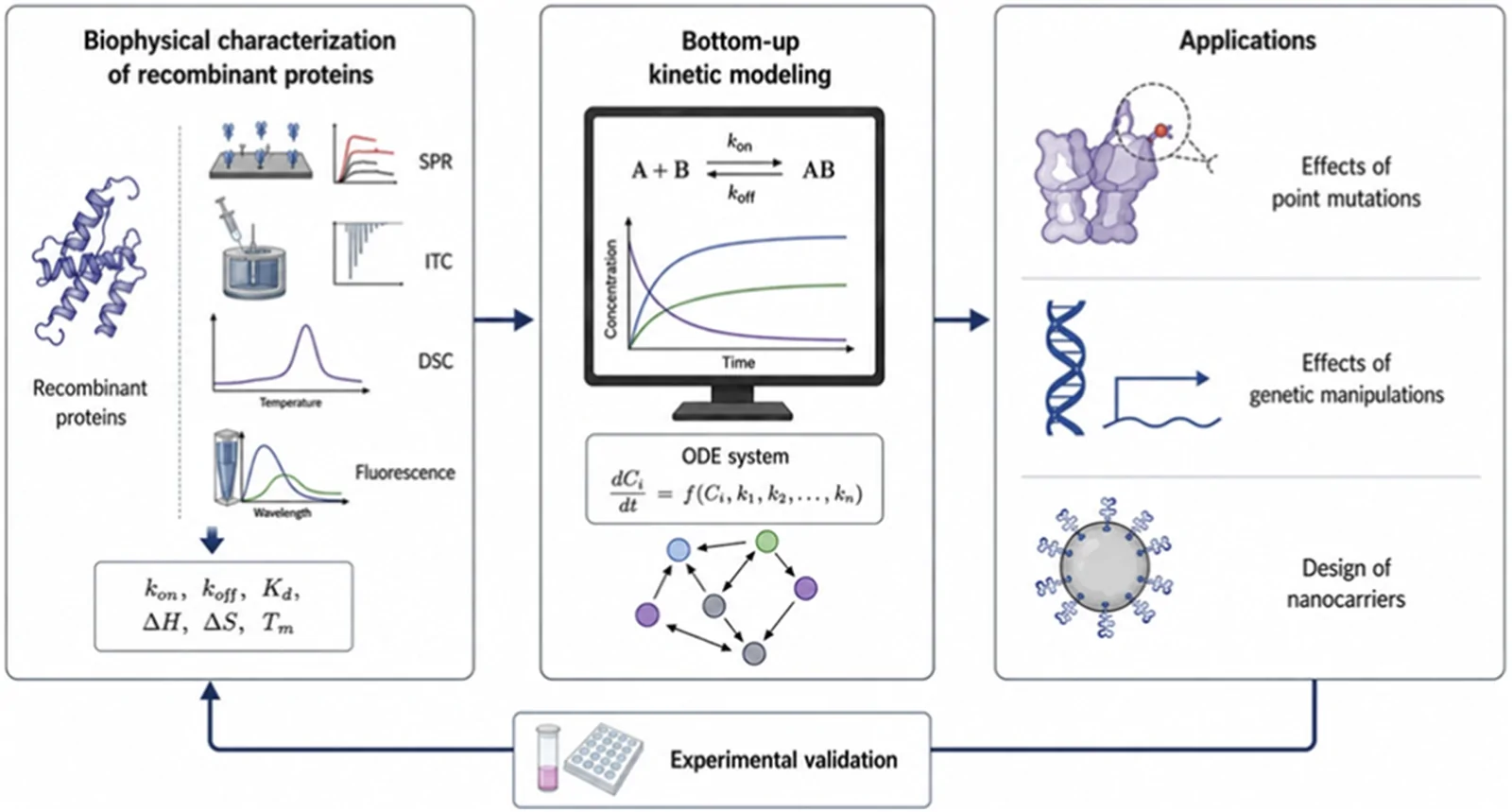
The integrated biophysical-computational workflow for recombinant protein research. Recombinant proteins are characterized using a battery of biophysical techniques—including Surface Plasmon Resonance (SPR), Isothermal Titration Calorimetry (ITC), Differential Scanning Calorimetry (DSC), and fluorescence spectroscopy—yielding kinetic and thermodynamic parameters (*k* ^*on*^, *k* ^*off*^ *,* K_d_, ΔH, ΔS, T_m_) that feed bottom-up kinetic models formulated as systems of ordinary differential equations (ODEs). The resulting computational framework enables quantitative predictions of the effects of point mutations and genetic manipulations on protein interaction networks, as exemplified by models of the vertebrate phototransduction cascade (Dell’Orco et al., 2009; Invergo et al., 2014; Beelen et al., 2021). The same approach can be extended to study the interaction of proteins with nanoparticles, predicting the time evolution and equilibrium composition of the protein corona (Dell’Orco et al., 2010b), with direct implications for the rational design of nanocarriers for biomedical applications. Experimental validation of model predictions closes the cycle, iteratively refining both the *in vitro* and *in silico* components of the workflow.

### Engineering of proteins to adapt them to specific or new functions

The first steps in protein design date back to the 1970s and 1980s, but with increasing computer power advanced algorithms allowed the *de novo* design of larger protein structures. Baker and colleagues designed protein structures using the Rosetta software (Yang et al., 2026; Bennett et al., 2023). The basic concept here uses small protein fragments stored in the Protein Data Bank as building blocks for larger units. Deep learning has since displaced fragment assembly. Generative models of protein backbones, such as those based on diffusion over structural coordinates, produce folds that satisfy a specified geometric or functional constraint. The constraint may be a binding site to be scaffolded, a symmetry to be imposed, or a metal centre to be housed. Inverse-folding networks then determine which sequence is most likely to adopt the generated backbone (Watson et al., 2023; Dauparas et al., 2022). The practical consequence is a change of regime. Binders against a chosen target, symmetric assemblies and enzyme active sites can now be designed in numbers, and the limiting step has moved from computation to experimental characterization. The maturity of the field was recognized by the 2024 Nobel Prize in Chemistry. One-half was awarded to David Baker for computational protein design, the other half jointly to Demis Hassabis and John Jumper for protein structure prediction. Unlike the 2012 prize discussed above, this award was given for computational rather than experimental work. It marks the point at which design and prediction became routine tools rather than specialist achievements. The field has developed to the design of advanced protein functions, for which the underlying structures were validated by crystallography and, increasingly, by cryo-EM. Expression and purification of designed proteins are therefore steps of primary importance for questions of protein dynamics, structural transitions and catalytic efficiency. They are also the only means by which a designed sequence is shown to be a protein at all, rather than a plausible string of amino acids.

## Conclusion

This tutorial guide addresses fundamental aspects of working with recombinant proteins and the use of heterologous expression systems. Virtually all areas of biological research face questions about physiological significance when results from *in vitro* studies are inspected or presented. We have set out the specific limitations inherent to heterologous expression systems. These are incomplete or non-native folding, missing or aberrant posttranslational modifications, the absence of native cofactors, and the loss of the crowded cellular environment. For each we have given the experimental strategies that address it. We have argued throughout that these limitations are properties to be measured rather than assumptions to be conceded.

The take-home message is not that recombinant proteins and native systems should be ranked. The goal is not to decide which is better, but to recognize that they answer different questions and that the answers are complementary. Reductionist experiments resolve mechanism at a level of detail that no measurement on a cell, a tissue or an organism can reach. Examples are an affinity, a rate constant, a thermodynamic driving force, an atomic structure, or the effect of a single substitution. Systemic experiments establish which of these mechanistic details matter in the context that ultimately defines biological function. Neither level is self-sufficient. Mechanistic parameters obtained with recombinant proteins are frequently what makes an observation in a cell interpretable at all. Conversely, a mechanism that predicts nothing at the cellular or organismal level has not yet been shown to be relevant. Several examples above make the point. One is the conformational switch in calcium sensor proteins. Another is the reconstruction of the phototransduction cascade from measured parameters. A third is the identification of downstream partners of cryptochrome in a species where no other approach was available. In each, progress came from integrating the two levels rather than from either one alone.

This integration is not only an academic matter. For much of drug discovery, *in vitro* starting points have been and will remain the mainstay. The purified target, its kinetic characterization and the structure of its complexes are the entry point from which most small-molecule programmes begin, and they have delivered. More than seventy protein kinase inhibitors have been approved since imatinib in 2001 (Cohen et al., 2021). Ribonucleotide reductase inhibitors remain in clinical use decades after the mechanism of the enzyme was worked out on purified material. Much of what is known about how enzymes work, and therefore about how they can be inhibited, descends from the mechanistic enzymology developed by Robert Abeles and his students. Their analysis of mechanism-based inactivation turned an understanding of catalysis into a strategy for designing inhibitors (Abeles and Maycock, 1976). That work was carried out on purified enzymes, and it is difficult to see how it could have been carried out otherwise.

*Science* marked its 125th anniversary in the special issue of 1 July 2005 by listing the most important open questions in research. One of them was how proteins find their partners. Protein-protein interactions are at the heart of life, and answering the question requires knowing more about the biochemistry and the structural organization of the cell (Kennedy and Norman, 2005). Two decades later the question is still open, and the observation still holds. Understanding how two proteins recognize each other in seconds, in the right orientation, inside a crowded cell, requires measured quantities: affinities, rate constants, structures, conformational populations. These are obtained on purified material, and they must then be tested where the process actually occurs. The methodological approaches described in this guide are the tools for the first half of that programme, and the reason they matter is the second half.
